# Ischemic proctitis after low‐dose‐rate brachytherapy using hydrogel spacer for prostate cancer

**DOI:** 10.1002/iju5.12444

**Published:** 2022-04-12

**Authors:** Ren Toriumi, Hiroshi Yaegashi, Takayuki Sakurai, Shigeyuki Takamatsu, Kazuyoshi Shigehara, Kouji Izumi, Yoshifumi Kadono, Atsushi Mizokami

**Affiliations:** ^1^ Departments of Integrative Cancer Therapy and Urology Kanazawa University Graduate School of Medical Science Kanazawa Ishikawa Japan; ^2^ Departments of Radiology Kanazawa University Graduate School of Medical Science Kanazawa Ishikawa Japan

**Keywords:** brachytherapy, hydrogels, ischemic colitis, proctitis, prostatic neoplasms

## Abstract

**Introduction:**

Recently, an absorbable hydrogel spacer is becoming more widespread to reduce rectal radiation dose for radiation therapy for localized prostate cancer.

**Case presentation:**

A 79‐year‐old male patient was referred to our hospital for radical treatment of organ‐confined prostate cancer. Low‐dose‐rate brachytherapy was performed, and the hydrogel spacer injection was added. The spacer was properly injected between the prostate and the rectum, causing no acute complications during hospitalization. Two months after low‐dose‐rate brachytherapy, the patient visited our hospital with constipation and melena, without fever. He was diagnosed with ischemic proctitis based on clinical courses and examinations. He was hospitalized for 19 days and made a complete recovery with conservative treatment.

**Conclusions:**

Herein, we report the first case of ischemic proctitis after low‐dose‐rate brachytherapy using hydrogel spacer for prostate cancer.

**Abbreviations & Acronyms**
ADT: androgen deprivation therapy
IP: ischemic proctitis
LDR: low‐dose‐rate
PSA: prostate‐specific antigen

**Keynote message**

Recently, the absorbable hydrogel spacer is becoming more widespread for radiation therapy for localized prostate cancer. We experienced a case of ischemic proctitis after low‐dose‐rate brachytherapy using hydrogel spacer. We should be aware of the possibility of ischemic proctitis as a hydrogel spacer complication.

## Introduction

Low‐dose‐rate (LDR) brachytherapy for organ‐confined prostate cancer is a well‐established method worldwide.^1^, ^2^, ^3^, ^4^, ^5^, ^6^

Recently, SpaceOAR system (Augmenix, Waltham, MA, USA), an absorbable hydrogel spacer, is becoming more widespread to reduce rectal radiation dose of radiation therapy for localized prostate cancer.^7^, ^8^, ^9^ However, rectal‐related complications have been reported to occur in the wrong indication by SpaceOAR although in a small number.^10^ Defecation control is one of the most important issues in radiotherapy because elderly patients often have potential defecation disorders.^11^ Herein we report a case of early postoperative ischemic proctitis (IP) after LDR brachytherapy using SpaceOAR. To our knowledge, this is the first report that describes IP as a complication of hydrogel spacer for prostate cancer.

## Case report

**Fig. 1:**
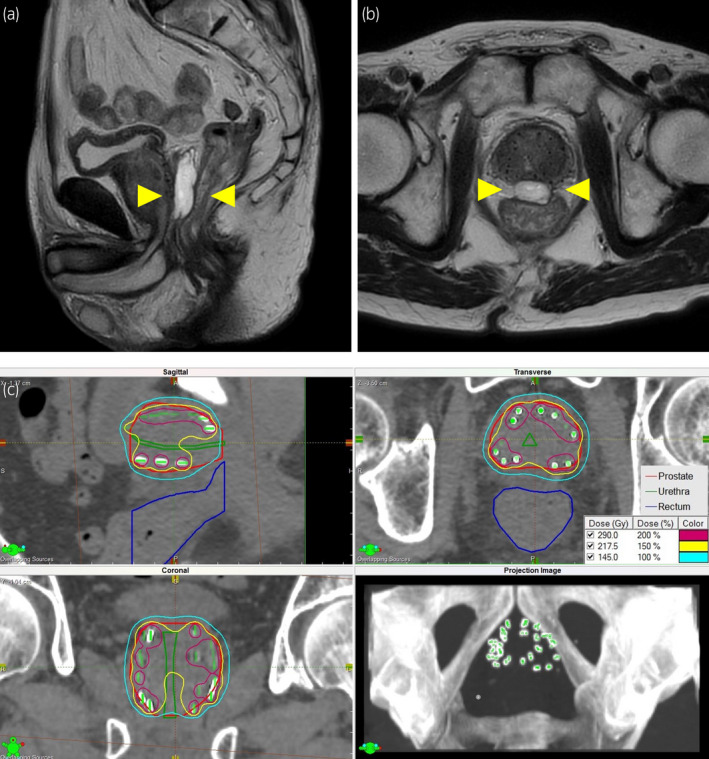
(a, b) Sagittal and transverse views of magnetic resonance imaging immediately after brachytherapy. The spacer (yellow arrowheads) was properly injected between the prostate and the rectum. (c) Isodose curve evaluation of prostate and rectum. The prostate is contoured in red, with 100% and 150% isodose lines in yellow and light blue, respectively. The rectum is contoured in blue. [Colour figure can be viewed at wileyonlinelibrary.com]

A 79‐year‐old male patient was referred to our hospital for radical treatment of organ‐confined prostate cancer. The initial prostate‐specific antigen (PSA) was 10.5 ng/mL, cT2aN0M0, Gleason score 3 + 4 = 7. He had a history of hemorrhoid surgery, but did not have an anal stenosis, defecation problems, and cardiovascular diseases. And he was not taking any medication. We planned LDR brachytherapy combined with 6 months of androgen deprivation therapy (ADT). After 3 months of neoadjuvant ADT, LDR brachytherapy was performed and 10 mL of hydrogel spacer was properly injected between the prostate and the rectum and outside the rectal serosa (Fig. 1), causing no acute complications during hospitalization. The prescribed radiation dose was 145 Gy and V100_prostate_ (the percentage of the prostate volume receiving 100% of the prescribed dose) was 99.24%; however, V100_rectum_ (the rectal volume receiving 100% of the prescribed dose) was 0.00 mL due to the spacer separating the rectum and the prostate by 11 mm (Table 1).

**Table 1:** Radiation dose

Prescribed dose (Gy)	145
Prostate	
D90 (Gy)	212.72
V100 (%)	99.24
V150 (%)	87.91
Urethra	
D30 (Gy)	229.69
V150 (cc)	0.48
Rectum	
V100 (cc)	0.00
V150 (cc)	0.00
D2cc (Gy)	72.45

Prostate D90: dose covering 90% of the prostate

Prostate V100: the percentage of the prostate volume receiving 100% of the prescribed dose

Urethra V150: the urethral volume receiving 150% of the prescribed dose

**Fig. 2:**
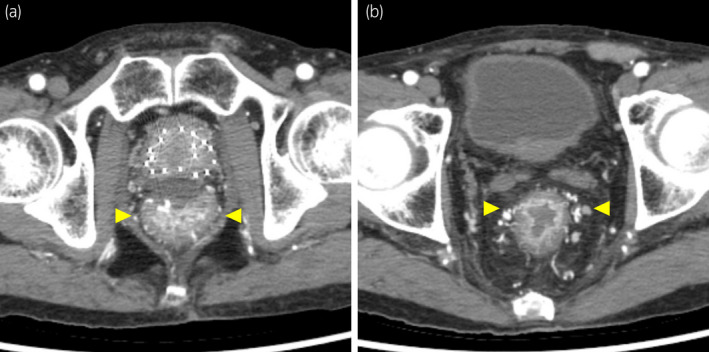
Contrast‐enhanced computed tomography at rehospitalization. (a) The spacer was still properly present. (b) Proctitis was suspected because of increased blood flow (yellow arrowheads) of the rectal mucosa. [Colour figure can be viewed at wileyonlinelibrary.com]

**Fig. 3:**
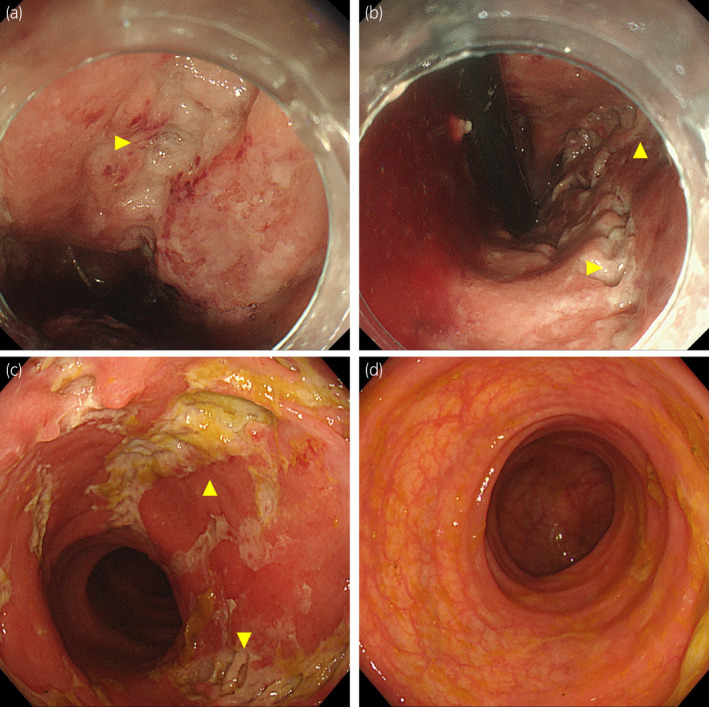
(a, b) Forward and retroflexion views of colonoscopy at rehospitalization. Colonoscopy revealed some longitudinal ulcers (yellow arrowheads) close to the anus. (c, d) Rectal and sigmoid views of colonoscopy 2 weeks after rehospitalization. The ulcers improved compared from the first time. No ulcers were noted in the sigmoid colon. [Colour figure can be viewed at wileyonlinelibrary.com]

Two months after the LDR brachytherapy, the patient visited our hospital with constipation and melena, without fever. Constipation lasted for 2 weeks before the visit but did not improve with laxatives. Contrast‐enhanced computed tomography indicated suspicious proctitis, and colonoscopy was performed for a definitive diagnosis (Fig. 2). Colonoscopy revealed some localized longitudinal rectal ulcers, without the point of hemorrhage (Fig. 3).

The stool culture upon hospitalization showed positive for *methicillin‐resistant Staphylococcus aureus* and *Escherichia coli*, and C‐reactive protein was high at 10.57 mg/dL and the serum procalcitonin was low at 0.06 ng/mL, excluding infectious disease as a diagnosis. We finally diagnosed him as IP based on clinical courses and examinations.

He was hospitalized and treated conservatively with fasting, and cefmetazole was administered to prevent secondary infection. Endoscopy was performed again 2 weeks after hospitalization, which revealed ulcer improvement (Fig. 3). The histopathological findings of rectal biopsy indicated active ulcer with necrotic tissue and negative for malignancy. The length of fasting, antimicrobial agent administration, and hospitalization were 8 days, 10 days, and 19 days, respectively. The PSA level at the second hospitalization was below sensitivity, indicating a well‐controlled prostate cancer.

## Discussion

We experienced a case of proctitis 2 months after LDR brachytherapy using hydrogel spacer, which was diagnosed as IP.

IP is a disease of ischemic colitis localized to the rectum. The most common site of ischemic colitis is the descending colon to the sigmoid colon, and the rectum is rarely reported.^12^ The patient with ischemic colitis presents abdominal pain (49.4%–73%) and diarrhea (14.2%–68%).^13^ The colonoscopy often reveals a longitudinal ulcerated or inflamed colon stripe.^14^ The present case is consistent with these clinical appearances.

One of the etiologies of ischemic colitis is reported to be transient mucosal ischemia of the colon.^12^, ^13^, ^14^ Teh *et al*. reported that one of the possible mechanisms of rectal injury with hydrogel spacer use is the ischemic injury based on the excessive tension from hydrogel spacer at the anterior rectal wall.^15^ Additionally, for patients with hemorrhoid, the most commonly demonstrated physiological abnormality is an increased resting anal pressure.^16^ In the present case, the spacer may have physically compressed the rectal mucosa, and further abdominal pressure due to constipation that lasted for 2 weeks may have triggered IP. Elderly people often have some kind of defecation disorder,^11^ and defecation should be properly controlled in patients undergoing radiotherapy.

IP diagnosis is difficult because it presents with nonspecific symptoms, and excluding differential diagnoses is necessary.^13^ First of all, we proposed some differential diagnoses – radiation, infectious, drug‐induced, or ischemic proctitis; colonic diverticulitis; and inflammatory bowel diseases such as Crohn's disease and ulcerative colitis. Drug‐induced proctitis and inflammatory bowel disease were excluded as a diagnosis because of the clinical course. Infectious proctitis was also excluded since the serum procalcitonin level was low and the lesion was located at the rectum only. Therefore, it was considered different from typical bacterial enteritis. Additionally, the absence of anal intercourse history ruled out proctitis due to sexually transmitted disease. Furthermore, a colonoscopy revealed no sign of colonic diverticulitis.

The possibility of radiation proctitis is a little higher. Acute radiation proctitis occurs during or within the first 3 months of radiation therapy, and patients usually present symptoms such as diarrhea, an urgency to defecate, tenesmus, cramping pain, and mild rectal bleeding.^17^ However, with the help of radiation oncologists and gastroenterologists, we diagnosed him as IP based on the low rectal radiation dose and more specific longitudinal ulcer findings in IP.^14^

Concerning prostate cancer treatments, hydrogel spacer is indicated not only in LDR brachytherapy but also in high‐dose‐rate brachytherapy or external beam radiotherapy.^18^ However, to our best of knowledge, no previous report has described IP after any method of radiotherapy with hydrogel spacer for prostate cancer. To prevent the adverse events after using hydrogel spacer, proper use of hydrogel spacer and defecation controls (e.g., perioperative use of laxatives) should be required.

## Conclusion

This is the first case report of IP after LDR brachytherapy for prostate cancer using hydrogel spacer worldwide. Physicians should pay attention to the possibility of IP as a complication of hydrogel spacer.

## Author Contributions

Ren Toriumi: Data curation; investigation; project administration; resources; visualization; writing – original draft; writing – review and editing. Hiroshi Yaegashi: Conceptualization; project administration; resources; writing – review and editing. Takayuki Sakurai: Data curation; investigation; resources; visualization; writing – original draft; writing – review and editing. Shigeyuki Takamatsu: Supervision; writing – review and editing. Kazuyoshi Shigehara: Writing – review and editing. Kouji Izumi: Writing – review and editing. Yoshifumi Kadono: Supervision; writing – review and editing. Atsushi Mizokami: Supervision; writing – review and editing.

## Conflict of interest

The authors declare no conflict of interest.

## Approval of the research protocol by an Institutional Reviewer Board

Not applicable.

## Informed consent

Not applicable.

## Registry and the registration no. of the study/trial

Not applicable.
